# Implementation of a Virtual Microphone Array to Obtain High Resolution Acoustic Images

**DOI:** 10.3390/s18010025

**Published:** 2017-12-23

**Authors:** Alberto Izquierdo, Juan J. Villacorta, Lara del Val, Luis Suárez, David Suárez

**Affiliations:** 1 Signal Theory and Communications Department, University of Valladolid, 47011 Valladolid, Spain; juavil@tel.uva.es; 2 Mechanical Engineering Area, Industrial Engineering School, University of Valladolid, 47011 Valladolid, Spain; lvalpue@eii.uva.es; 3 Civil Engineering Department, Superior Technical College, University of Burgos, 09001 Burgos, Spain; luis.a.suarez.vivar@gmail.com; 4 Dyquo Company, University of Burgos, 09001 Burgos, Spain; dave@dyquo.com

**Keywords:** virtual array, MEMS microphones, high resolution acoustic images

## Abstract

Using arrays with digital MEMS (Micro-Electro-Mechanical System) microphones and FPGA-based (Field Programmable Gate Array) acquisition/processing systems allows building systems with hundreds of sensors at a reduced cost. The problem arises when systems with thousands of sensors are needed. This work analyzes the implementation and performance of a virtual array with 6400 (80 × 80) MEMS microphones. This virtual array is implemented by changing the position of a physical array of 64 (8 × 8) microphones in a grid with 10 × 10 positions, using a 2D positioning system. This virtual array obtains an array spatial aperture of 1 × 1 m^2^. Based on the SODAR (SOund Detection And Ranging) principle, the measured beampattern and the focusing capacity of the virtual array have been analyzed, since beamforming algorithms assume to be working with spherical waves, due to the large dimensions of the array in comparison with the distance between the target (a mannequin) and the array. Finally, the acoustic images of the mannequin, obtained for different frequency and range values, have been obtained, showing high angular resolutions and the possibility to identify different parts of the body of the mannequin.

## 1. Introduction

An array is an arranged set of identical sensors, fed in a specific manner. The array beampattern can be controlled by modifying the geometry of the array (linear, planar, etc.), the sensor spacing, and the response, the amplitude and the phase excitation of each sensor [1]. Microphone arrays are a particular case of these systems. They are used in applications such as speech processing, echo cancellation, localization and sound sources separation [2]. By using beamforming techniques [3], the array beampattern, particularly its mainlobe, can be electronically steered to different spatial positions, allowing spatial filtering, i.e., the discrimination of acoustic sources based on their position. Array resolution depends on the mainlobe width of its beampattern, which is directly related with the spatial aperture of the whole array, i.e., its maximum dimension. This spatial aperture is also related in time with the number of sensors which form the array. Array mainlobe narrows when the spatial aperture of the array increases, which also increases with the number of sensors.

The authors of this paper have experience in the design and development of acoustic ULAs (Uniform Linear Arrays) [4,5,6,7,8]. These arrays are simple, but they are limited to estimate the spatial position of the sound source in only one dimension (azimuth or elevation). To obtain spatial information in two dimensions, working with planar arrays, with sensors distributed on a surface, is necessary. Working with planar arrays leads to an increase in system complexity and in the space required by the acoustic sensors and the associated hardware. The extension from a 1D to a 2D array increases the number of channels required exponentially. This increment is directly related with the complexity and the cost of the system.

The acronym MEMS (Micro-Electro-Mechanical System) refers to mechanical systems with a dimension smaller than 1 mm, which are manufactured with tools and technology arising from the integrated circuits (ICs) field. These systems are mainly used for the miniaturization of mechanical sensors [9]. The application of MEMS technology to acoustic sensors has allowed the development of high-quality microphones with high SNR (Signal to Noise Ratio), low power consumption and high sensitivity [10].

A typical acquisition and processing system for acoustic arrays, based on analog microphones, has four basic elements: sensors, signal conditioners, acquisition devices and signal processor. Digital MEMS microphones include a microphone, a signal conditioner and an acquisition device incorporated in the chip itself. For this reason, an acquisition and processing system for an acoustic array, based on MEMS microphones, is reduced to two basic elements: MEMS microphone and a processing system. The integration of the microphone preamplifier and the ADC in a single chip significantly reduces costs and the space occupied by the system. These features allow building arrays of high dimensions, with a high number of sensors, and consequently with a narrow mainlobe, which means a good array resolution. These characteristics of MEMS microphones, together with the characteristics of planar arrays, were joined in the last system developed by the authors. This system was based on a planar array of digital MEMS microphones [11,12].

In recent years, techniques for obtaining acoustic images have been developed greatly and rapidly. At present, acoustic images are associated with a wide variety of applications, such as non-destructive testing of materials, medical imaging, underwater imaging, SONAR (SOund Navigation and Ranging), geophysical exploration, machinery health monitoring, etc. [13]. Arrays of MEMS microphones are specially designed for acoustic source localization [14]; however, they are also used in other applications such as speech processing [15], turbulence measurements [16], identifying geometric dimensions and internal defects of concrete structures [17], or acoustic imaging [18,19]. Due to the high diversity of the applications of these arrays, the authors widened these uses to another field, such as the industrial one [20]. Working with machinery has the disadvantage that the reverberation of typical industrial plants could be really important unless a sufficient number of microphones in the array are used [21].

Although MEMS technology allows building arrays of high dimensions, the total number of needed sensors could be so high that the implementation of such an array would be economically unfeasible. The large microphone arrays which have been built have only several hundreds of sensors. Among these arrays, the LOUD array [22]—with 1020 sensors—and the Sorama array [19]—with 1024 sensors—stand out. Building arrays with high dimensions is associated with problems related with the mechanical stiffness of the system and with the synchronization of the captured signals. Thus, building virtual arrays by means of moving a small physical array with a positioning system is usual [23]. The problem with these solutions is that the capture time is too high, because it is necessary to repeat the capture for each of the selected positions. One solution to this problem can be the implementation of 2D virtual arrays using only one sensor that moves by means of a 2D positioning system [24,25], or using a linear (1D) array that moves by means of a 1D positioning system [26,27,28]. These systems used to be built with analogic sensors and high-cost analogic acquisition systems.

This paper shows a mixed model. It shows the simulation and the analysis of a virtual 2D array of high dimensions—6400 MEMS (80 × 80) microphones—by means of a 2D positioning system. This positioning system places a 2D physical array of 64 MEMS microphones (8 × 8) [11] in a grid with 10 × 10 positions. This technique allows building systems with noticeable reduced acquisition time, compared with the systems that move only one sensor or a 1D linear array. The technique also allows building systems with thousands of sensors with reduced cost, by using the low-cost MEMS technology.

The designed system is based on the SODAR (SOund Detection And Ranging) principle. Although LIDAR principle could also be used to obtain images of the targets, SODARs have its own advantages. SODAR advantages are associated to the use of sound instead of light. Thus, using a SODAR system is useful in those environments where the conditions to light propagation are unfavorable, such as environments with smoke, fog, etc., or when the object to be detected is very scattering.

Section 2 introduces the system and methods used in this study, that is the processing and acquisition system, based on a planar array of MEMS microphones, the 3D positioning system, and the methodology to implement the virtual array. Section 3 presents the results obtained by using this system to acquire acoustic images of a mannequin, as well as future research lines. Finally, Section 4 contains the conclusions which authors have drawn based on the obtained results.

## 2. Material and Methods

### 2.1. Processing and Acquisition System

This section shows the acquisition and processing system, based on a 2D array of MEMS microphones and the 3D positioning system used in this study.

**Figure 1:**
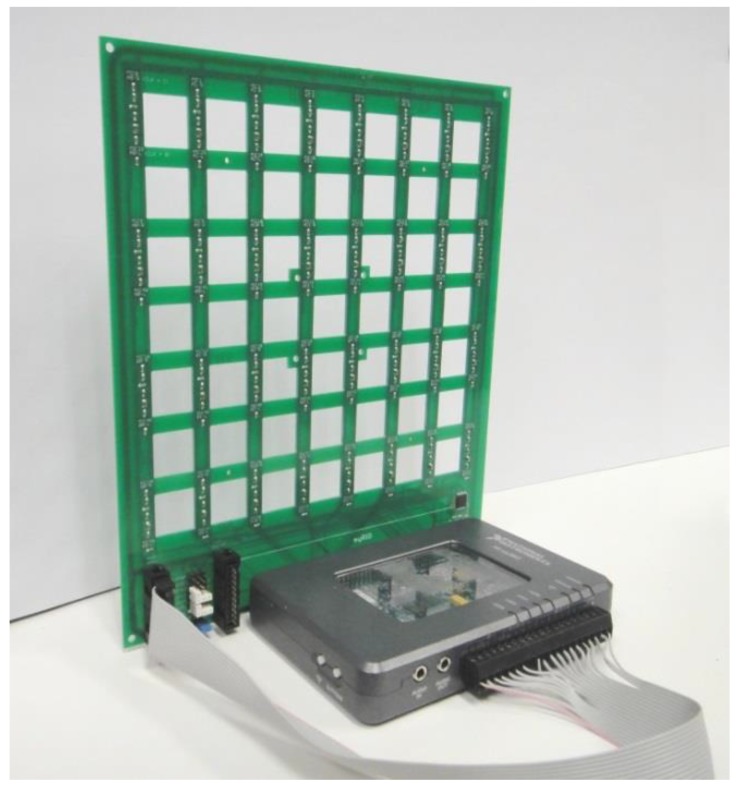
Array module with myRIO and MEMS array board.

The acoustic images acquisition system used in this paper is based on a Uniform Planar Array (UPA) of MEMS microphones. This array, which has been entirely developed by the authors, is a square array of 64 (8 × 8) MEMS microphones that are uniformly spaced every 2.5 cm in a rectangular Printed Circuit Board (PCB), as shown in Figure 1.

This array was designed to work in an acoustic frequency range between 4 and 16 kHz. The 2.5 cm spacing corresponds to λ/2 for the 8 kHz frequency. This spacing allows a good resolution for low frequencies, while avoiding grating lobes for high frequencies in the angular exploration zone of interest.

For the implementation of this array, MP34DT01 microphones of STMicroelectronics—digital MEMS microphones with PDM interface—were chosen, with the following features: low-power, omnidirectional response, 63 dB SNR, high sensitivity (−26 dBFS) and a nearly flat frequency response (±6 dB in the range of 20 Hz to 20 kHz).

**Figure 2:**
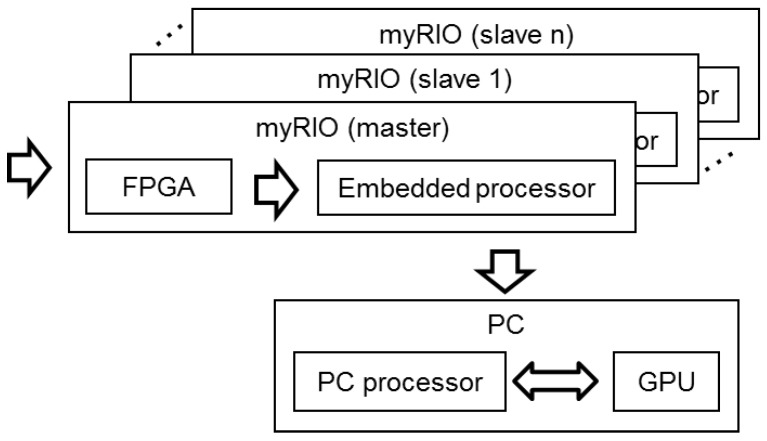
Global hardware setup.

A MyRIO platform [9] is the base unit for this system. This platform belongs to the Reconfigurable Input-Output (RIO) family of devices from National Instruments, which is oriented to sensors with nonstandard acquisition procedures. The embedded processor included in myRIO can run all software algorithms to generate acoustic images, so it can be used as a standalone array module formed by a myRIO connected to a MEMS array board, as shown in Figure 1. Although myRIO can work as a standalone system, the lack of display means that it is usually controlled from a PC connected using a Wi-Fi interface. In a global hardware setup, as shown in Figure 2, the system includes a PC and one or more array modules.

**Figure 3:**
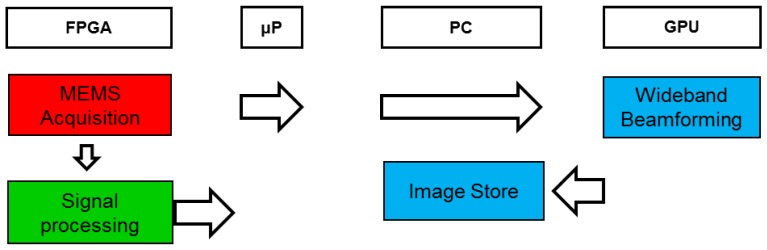
System framework.

The algorithms implemented in the system, shown in Figure 3, can be divided into three blocks: MEMS acquisition, signal processing and image generation (wideband beamforming and image storage). The programming language used is LabVIEW 2015, along with its Real Time, FPGA, and Graphical Processing Unit (GPU) modules, which allows developing applications on different hardware platforms such as those used in the system: FPGA, Embedded Processor (EP), PC, and GPU.

- In the acquisition block, each MEMS microphone with a PDM interface performs signal acquisition.
- In the signal processing block, two routines are implemented: deinterlacing and decimate and filtering, obtaining 64 independent signals (one of each MEMS of the array).
- Finally, in the image generation block, based on wideband beamforming, a set of N × N steering directions are defined, and the beamformer output are assessed for each of these steering directions. The images generated are then displayed and stored in the system.

A processing platform, enabled to work at a low level—to acquire signals—and at a high level—to implement spatial processing algorithms—using low cost commercial systems, was defined. The processing algorithms are shared between the FPGA and the PC, excluding beamforming, which is implemented on the GPU, as shown in Figure 3. The embedded processor is used to control and transfer data between the PC and FPGA.

### 2.2. Positioning System

**Figure 4:**
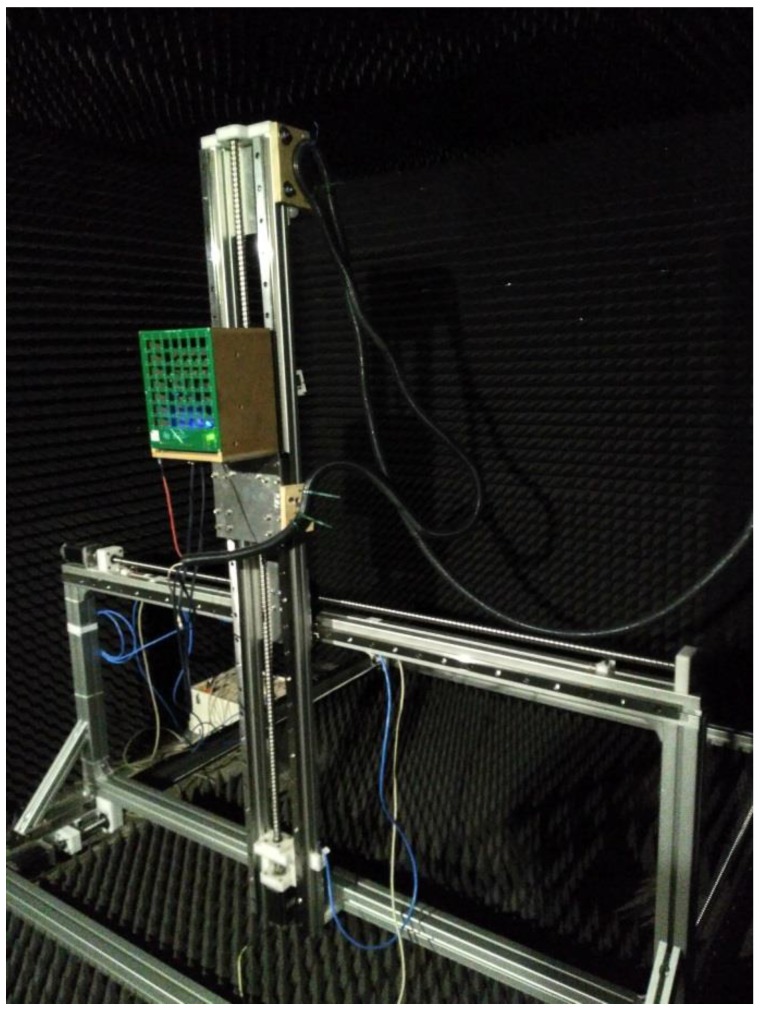
3D positioning system.

The array module is moved in space with a Cartesian 3D positioning system, capable of positioning the array inside a cubic volume of 1500 × 1500 × 1500 mm^3^ with repeatability accuracy of 0.02 mm in each spatial dimension. It is shown in Figure 4. While it is a 3D positioner, only two dimensions are being used in this application.

### 2.3. Virtual Array Principle

The explained 8 × 8 planar array of MEMS microphones is placed on a 2D positioning system, as can be observed in Figure 4. To obtain the virtual array, the position of this 8 × 8 array is changed on the vertical and horizontal directions, moving it on a surface that is parallel to the array itself. The array obtains an acoustic image of the target under test in each of these positions.

The data acquired by the 8 × 8 array in each of the positions are added in a data structure, equivalent to the one that would be obtained with an array of hundreds or thousands of sensors. These data is spatially processed, using a beamforming algorithm, to obtain the high resolution acoustic images.

In this work, a virtual array has been developed by moving the single 8 × 8 array on a grid of 10 × 10 positions, using two different steps in both directions:

- Even steps: 1.25 cm, which is the half sensor spacing of the 8 × 8 array.
- Odd steps: 10 cm, which is the spatial aperture of the 8 × 8 array.

**Figure 5:**
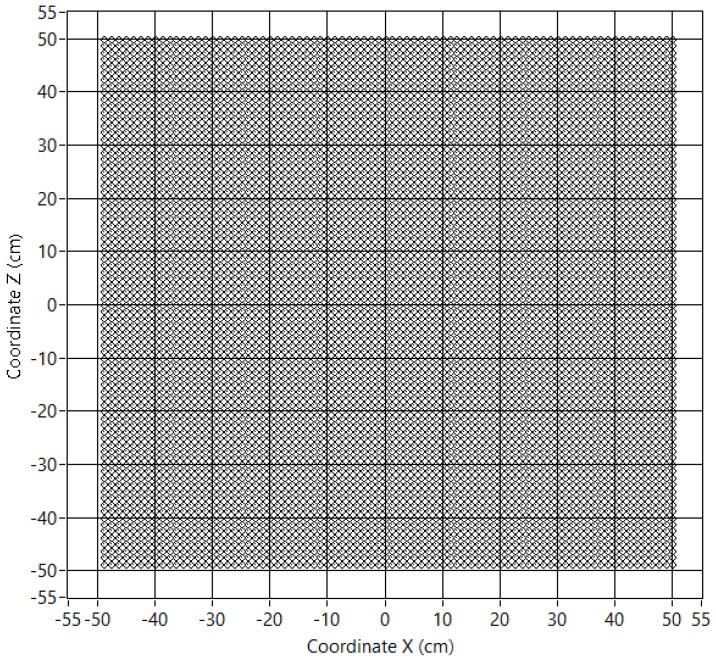
Virtual array sensor positions.

With this procedure, an array of 80 × 80 microphones with uniform 1.25 cm spacing has been developed. Table 1 shows the characteristics of this virtual array, and Figure 5 shows the sensor positions of the virtual array.

**Figure 6:**
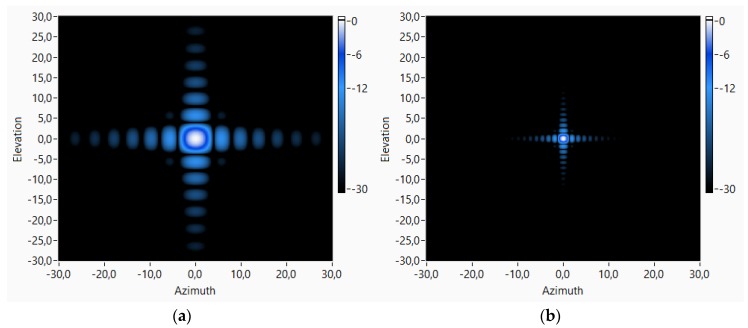
The 80 × 80 array theoretical beam pattern, pointing to (0°, 0°) direction, for working frequencies: (**a**) 4 kHz; and (**b**) 15 kHz.

An array of 80 × 80 sensors, uniformly spaced at 1.25 cm, has a high spatial resolution, as can be observed in Figure 6. Figure 6 shows the theoretical beam pattern of this 80 × 80 array working at two different frequencies, 4 and 15 kHz, pointing to the broadside (azimuth = 0° and elevation = 0°). For these frequencies, it can be observed that the main lobe widths of the beam patterns are 4.8° and 1.6°, respectively.

**Figure 7:**
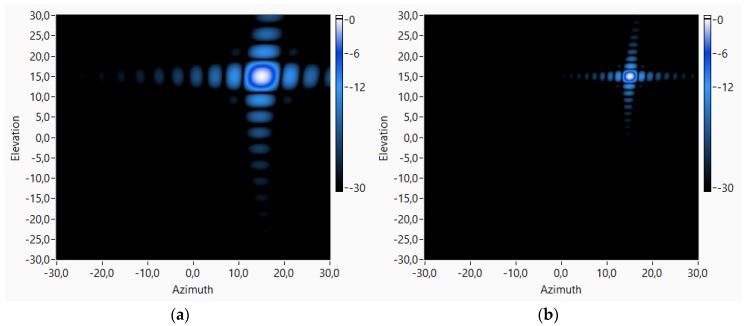
The 80 × 80 array theoretical beam pattern, pointing to (15°, 15°) direction, for working frequencies: (**a**) 4 kHz; and (**b**) 15 kHz.

The tests done with this 80 × 80 virtual array have been carried out inside a hemianechoic chamber with a 5 × 3 × 2.5 m^3^ working area. The problem of working with such a large array inside this hemianechoic chamber is that the plane wave assumption is not reasonable because of the ratio between the array spatial aperture and the distance between the target and the array. The array is too close to the target and the acoustic waves are spherical, as can be observed in Figure 7. Figure 7 shows that this effect is clearer if the array is pointing to directions away from the broadside.

**Figure 8:**
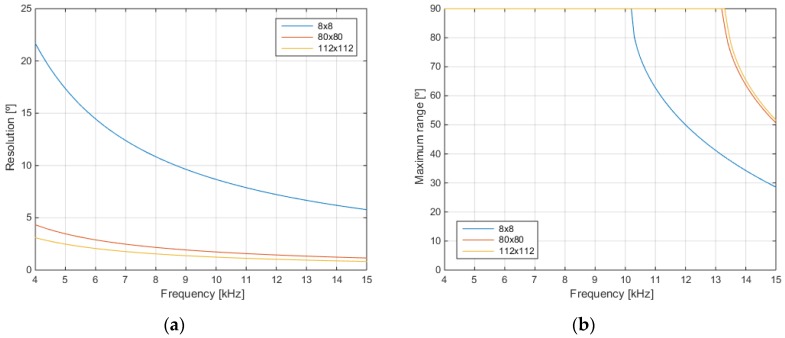
(**a**) Resolution; and (**b**) maximum range vs. frequency for physical array 8 × 8 and 80 × 80 and 112 × 112 virtual arrays.

Spatial resolution of the virtual array depends linearly on the array spatial aperture and it is inversely proportional to the working frequency [29], as shown in Figure 8a. In this work, a virtual array with a size of 1 m × 1 m that works with a maximum frequency of 15 kHz has been used. Under these conditions, the obtained spatial/angular resolution is 1.6° for this virtual array. This value is smaller than the resolution of the physical 8 × 8 array, which is 6.4°. In the developed system, where the positioning system has a maximum length of 1.5 m (112 × 112 sensors), the maximum spatial resolution that could be obtained would be 1.1°.

The dynamic range in these systems, that is, the maximum working angle, is fixed by the presence of grating lobes (spatial aliasing) [1,2]. Using this virtual array, sensor spacing is halved, so the dynamic range increases if it is compared with the physical array. Figure 8b shows that the maximum range of the 8 × 8 physical array is ±90° for working frequencies below 10 kHz and it decreases to ±28° at the maximum working frequency of 15 kHz. Using the virtual array, these limits increase up to 13 kHz for the maximum range of ±90°, and to ±51° for the maximum working frequency of 15 kHz.

**Figure 9:**
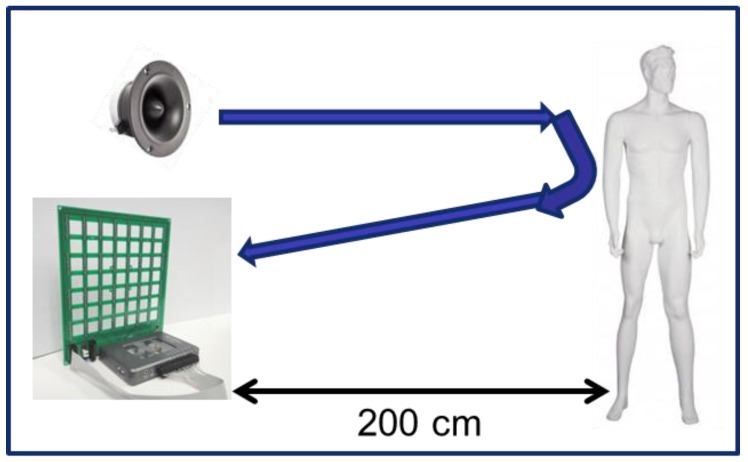
SONAR (SOund Detection And Ranging) principle.

After this theoretical analysis of the 80 × 80 array, this virtual has been used to obtain acoustic images of a mannequin, by using the SODAR principle shown in Figure 9: a tweeter generates a known sound signal towards the mannequin under test; this signal is reflected over it; and the microphones of the array receive the reflected signal. Finally, the signal that is received by the array is used to obtain the acoustic images of the mannequin.

## 3. Results and Discussion

### 3.1. Virtual Array Performance

**Figure 10:**
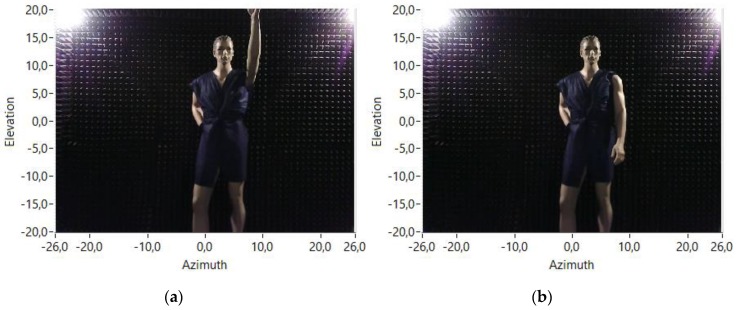
Mannequin positions: (**a**) arm up; and (**b**) arm down.

Some tests have been carried out with a mannequin placed on a fixed point inside the anechoic chamber, 240 cm opposite the array. This mannequin has been placed in two different positions: one with the arm up, as can be observed in Figure 10a, and the other one with the arm down, as shown in Figure 10b. The working frequency of the system for these tests is 15 kHz.

**Figure 11:**
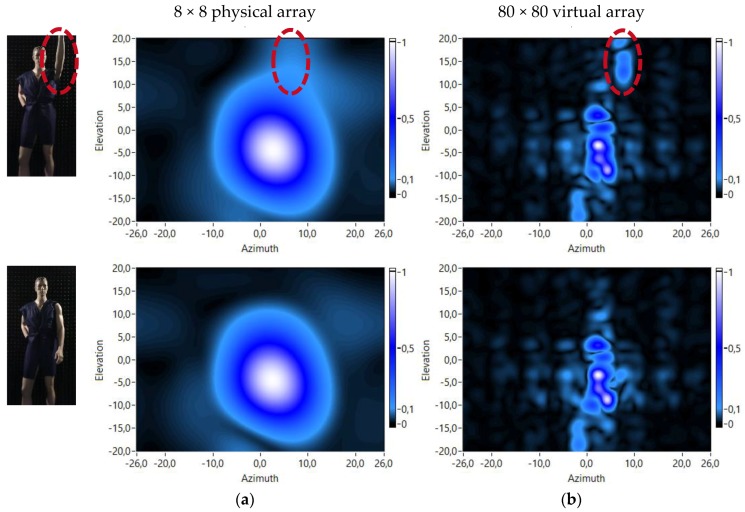
Array responses: (**a**) 8 × 8 physical array; and (**b**) 80 × 80 virtual array.

Acoustic images of the mannequin in the two different positions have been obtained using the physical 8 × 8 array and also using the 80 × 80 virtual array to see the different performance of both arrays. The obtained acoustic images using both arrays and both mannequin positions are shown in Figure 11. In the acoustic images of the mannequin with the arm up, the response of this arm to the transmitted acoustic signal can be noticed. These responses are highlighted with a red ellipse on the corresponding images of Figure 11.

These figures show that the acoustic images obtained with the 80 × 80 virtual array (Figure 11b) have a higher resolution than the acoustic images obtained with the 8 × 8 physical array (Figure 11a), due to its larger “virtual” spatial aperture.

### 3.2. Analysis of Frequency and Range Dimensions

Once the higher resolution of the virtual array has been verified, more tests have been carried out to analyze the influence of changes in the frequency and the range dimensions, on the array resolution.

#### 3.2.1. Frequency Analysis

**Figure 12:**
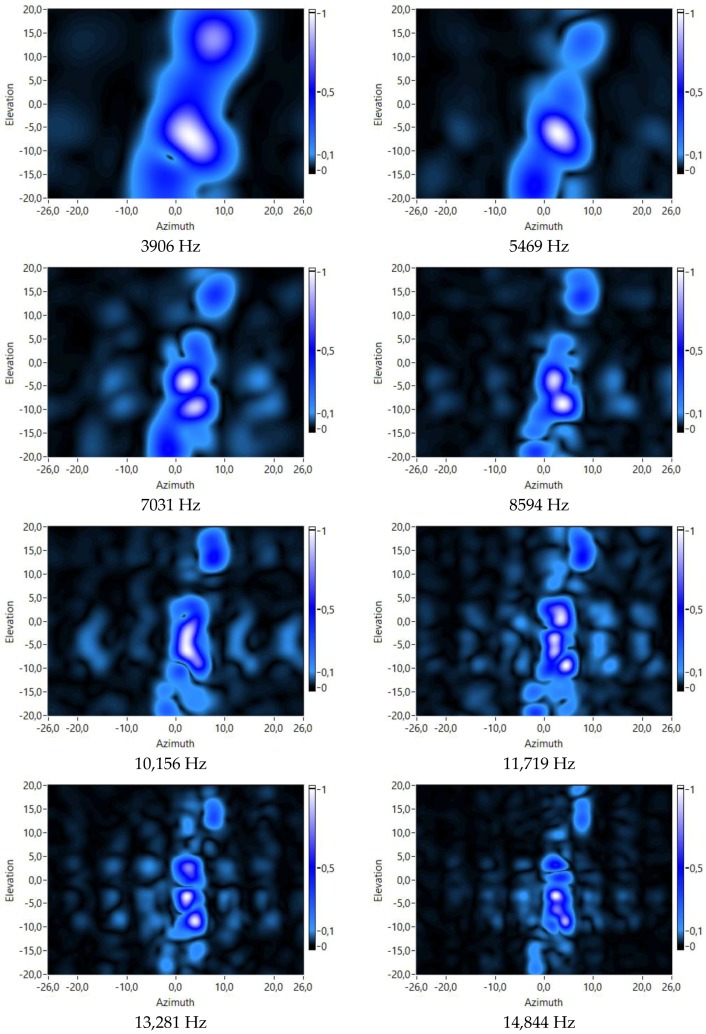
Virtual array responses for several frequencies and a range of 240 cm.

First, some tests have been carried out with a fixed value of the range dimension to 240 cm. In these tests, the value of the frequency dimension has varied for each test, obtaining different acoustic images of the same target (the mannequin). These acoustic images are shown in Figure 12.

Analyzing these figures, it is observed that, as expected, if frequency value increases, the resolution of the acoustic image also increases.

#### 3.2.2. Range Analysis

**Figure 13:**
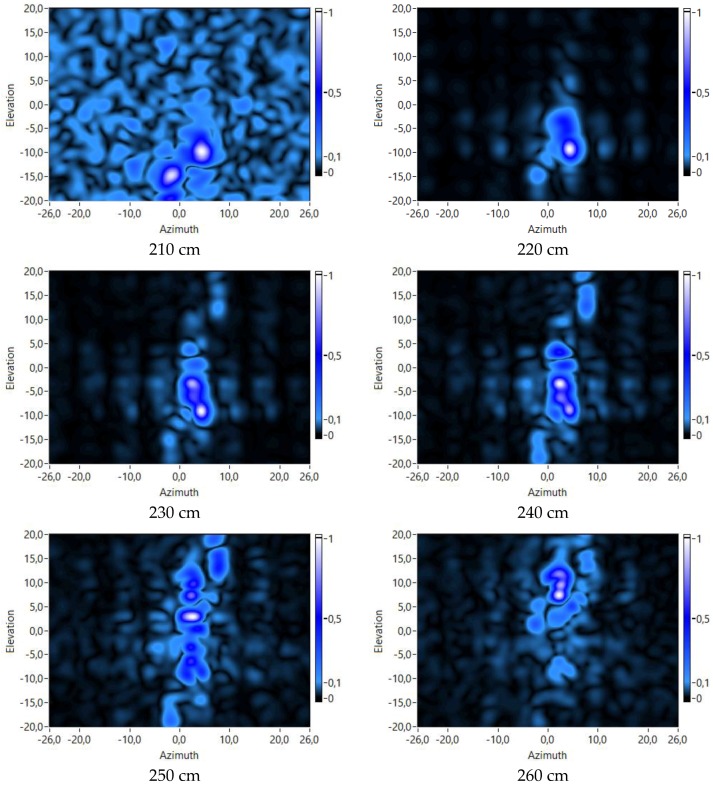
Virtual array responses for several ranges and a frequency of 13 kHz.

The next tests have been carried out with a fixed value of the frequency dimension to 13 kHz. In these tests, the value of the range dimension has varied for each one, also obtaining different acoustic images of the mannequin. These acoustic images are shown in Figure 13.

An analysis of Figure 13 shows that different parts of the body of the mannequin can be identified at different distances from the array. For example, images in Figure 13 show that:

- The legs of the mannequin are a bit closer to the array than its torso. It can be observed that, at low ranges (210–220 cm), the corresponding acoustic images show maxima at lower values of the elevation dimension, which correspond with the position of the legs. At higher values (230–240 cm), the maximum values are at higher elevation values, which correspond with the position of the torso.
- The head of the mannequin is the part of the body that is farther from the array. It can be observed that at higher ranges (250–260 cm), the maxima are shown at high elevation values, which correspond with the position of the head.
- It can also be observed that the head is even farther from the array than the arm that is raised.

These high resolution acoustic images could be used to identify people in biometric acoustic systems. Thus, a deep analysis of the acoustic images would be useful to extract representative parameters in their four dimensions, i.e., azimuth, elevation, frequency and range, and also to obtain which of these dimensions are more representative in the identification tasks.

## 4. Conclusions

A virtual array of 80 × 80 MEMS microphones has been developed moving a single 8 × 8 planar array of MEMS microphones on a grid of 10 × 10 positions, by means of a 2D positioning system. The developed virtual array has been tested by using it to obtain acoustic images of a mannequin in two different positions, using the system as an active SODAR.

The objective of this virtual array is to obtain a system with the performance of a system formed by thousands of sensors, but working only with dozens of them. A large array is associated to a high spatial aperture, and therefore to a high spatial resolution. This increment in the spatial resolution using such a virtual array allows obtaining high resolution acoustic images.

Analyzing the dependence of the acoustic images with their frequency and range dimensions it can be observed that: (i) there is an increment in the spatial resolution of the acoustic images with the frequency; and (ii) different parts of the body of the mannequin can be identified at different distances/ranges from the array.

## Figures and Tables

**Table 1:** Virtual array characteristics.

Sensor Positions	Number of Sensors	Area	Dimensions
80 × 80	6400	1 m^2^	1 m × 1 m
